# Joint optimization of overbooking and seat allocation for high-speed railways considering stochastic demand

**DOI:** 10.1371/journal.pone.0312745

**Published:** 2024-11-18

**Authors:** Jing Xu, Lianbo Deng, Xinlei Hu, Jiayi Liu, Weidong Tan

**Affiliations:** School of Traffic and Transportation Engineering, Central South University, Changsha, Hunan, China; Chang’an University, CHINA

## Abstract

To mitigate empty seat loss caused by random passenger no-show behavior, this study extends seat allocation to joint optimization of overbooking and seat allocation for high-speed railways (HSR). Assuming that stochastic passenger demand follows a specific distribution and considering various constraints, including train capacity, demand, and denied boarding rate constraints, a nonlinear stochastic programming model for joint optimization of overbooking and seat allocation for HSR is constructed with the aim of maximizing railway expected revenue. To solve this optimization model, a multi-level optimization algorithm is designed. Based on the sampling averaging approximation method, demand scenarios and passenger no-show scenarios are generated and the optimization problem is decomposed, including the joint optimization of overbooking and seat allocation under a single demand scenario, and the ticket adjustment under other demand scenarios. For the former, it is further divided into two sub-problems according to the stochastic nature of passenger no-show behavior, which is optimized iteratively. Finally, the effectiveness of the proposed model and algorithm is evaluated through numerical studies. The results demonstrate that the proposed joint optimization method effectively addresses the randomness of passenger demand and no-show behavior, thereby improving HSR expected revenue and making up for the empty seat loss resulting from passenger no-show behavior.

## 1 Introduction

In recent years, China has made significant achievements in enhancing its railway network and passenger travel demand has substantially increased. The operating mileage of China’s railway network has reached approximately 159,000 kilometers by the end of 2023, including 45,000 kilometers of high-speed railway (HSR) [1]. In 2023, China’s railway sector handled a record-high 3.68 billion passenger trips [2]. However, the expansion of railway transportation capacity has struggled to keep up with the robust growth in passenger travel demand, leading to a significant contradiction between capacity and demand. Consequently, the optimization of seat allocation and ticket sales organization has become a prominent topic. To address this challenge, China’s railway system formulates ticket sales strategies and allocates tickets for each origin-destination pair (OD) based on historical passenger flows and ticket pre-sale information as much as possible to facilitate passengers’ convenient travel and maximize the utilization of railway capacity resources.

The currently widely implemented ticketing organization strategies in China include ticket pre-allocation, seat reuse, OD-shared tickets, adjustment of restricted selling sections and ticket dynamic optimization. In addition, innovative ticket system products such as “counting tickets” and “fixed-term tickets” have been proposed to enhance convenience for passengers. Therefore, the scientific and intelligent seat allocation has attracted more research attention [3–10]. For example, Zhu et al. [3] studied the dynamic seat allocation problem during HSR ticket sales and proposed a novel bidding price control strategy based on the maximum sequence principle. Yan et al. [5] investigated the impact of flexible train compositions on seat control and developed a stochastic nonlinear programming model considering stochastic demand and passenger choice behavior to make seat allocation and train composition decisions. Zhao et al. [10] studied revenue management for homogeneous seats of high-speed trains and aimed to optimize the number of tickets for different price levels by setting multiple price levels based on passenger preferences.

While these studies contribute to optimizing railway capacity allocation, improving passenger transportation supply and meeting passenger travel demand, they often ignore the stochastic nature of passenger demand and no-show behavior. Passenger demand forms the foundation for seat allocation and its random characteristics introduce complexity to this process. Moreover, passengers may refund tickets or give up boarding the train after purchasing tickets due to personal itinerary changes, bad weather, or traffic delays, which is regarded as passenger no-show behavior. Passenger no-show behavior generally occurs close to the departure time, resulting in empty seat loss, challenges in reselling tickets, and inefficient capacity utilization. The world’s largest ticketing transaction system, the 12036 ticketing system, has played a crucial role in facilitating ticket purchasing. The system offers 24-hour internet ticket refund services and extends refund windows until just before departure, which provides great flexibility and convenience for passengers refunding tickets. Consequently, focused on the stochastic nature of passenger no-show behavior and demand, studying how to mitigate empty seat loss and increase seat occupancy rates holds both theoretical significance and practical value.

Overbooking strategy for HSR selling more tickets than the train’s transportation capacity can make up for empty seat loss to some extent. However, due to fixed train seating capacity, excessive overbooking may result in the number of boarding passengers exceeding the seating capacity. In such cases, some passengers will be denied boarding (DB) and compensated, leading to DB loss for railway operators. On the other hand, insufficient overbooking may not effectively address the stochastic nature of passenger no-show behavior. Overbooking strategy is widely used in various industries, including aviation, railways, hotels, healthcare, and container shipping [11–16]. In 2021, the U.S. Department of Transportation increased the minimum cash compensation amount for involuntary DB passengers by airlines and prohibited airlines from denying boarding to passengers who have already boarded the aircraft [17]. In the aviation industry, overbooking strategies have been extensively studied [16,18–20]. Fard et al. [16] used dynamic programming to study the overbooking problem in the aviation industry aiming to maximize airline market share. Dalalah et al. [21] developed a voluntary overbooking model under rational expectations to promote cooperation between passengers and airlines with the aim of maximizing the expected revenue for airlines and employed the decision tree analysis method. Overbooking is also a type of seat management, and therefore, some researchers have integrated overbooking with seat allocation to accommodate more passengers and reduce seat vacancy [22,23]. Lan et al. [23] studied the airline revenue management problem for a single leg by integrating expected overbooking costs and revenue and utilized a robust optimization approach based on the regret criterion.

Due to the complex operational characteristics involving multiple trains, ODs and legs, and complicated capacity utilization relationships in the railway industry, the rich overbooking research experiences in aviation with relatively straightforward operational mode cannot be directly applied to the railway industry. Specifically, in the aviation industry, a flight generally travels between one single OD and its whole flight capacity is allocated to this OD; a flight may serve three ODs with two ODs sharing the flight capacity on each leg when it is a stopover flight. The number of legs and the number of ODs are extremely limited and the relationships between flight capacity and ODs are relatively straightforward. The correspondence between the overbooking tickets and ODs is clear. However, in HSR, there are multiple trains running parallel along the HSR line, each of which serves multiple stations and ODs. Multiple ODs share the train capacity of each leg, which is the segment between two consecutive stops for a train, causing intricate interactions between trains, legs, and ODs. Overbooking in HSR is reflected in the sold tickets beyond the number of seats in a certain leg, and the overbooking tickets can be utilized by multiple ODs in competition. Compared with aviation, HSR trains have more stop stations, the number of ODs is relatively large, and the capacity sharing relationship for HSR is more complicated. Multi-train and multi-stop characteristics for HSR make the joint optimization of overbooking and seat allocation for HSR considering stochastic demand quite different from that for airline. Therefore, studying RM strategies that work for HSR is essential.

In the railway in Japan and Western Europe where ticket and seat are separated, overbooking is utilized. However, tickets and seats are one-to-one corresponding in China, leading to challenges in implementing overbooking. Current research on overbooking in the railway primarily remains at the theoretical level, lacking quantitative investigations [24]. For instance, Yan et al. [25] established an overbooking optimization model considering the economic and social benefits of China’s HSR network and conducted numerical calculations via MATLAB software. Sato and Sawaki [26] investigated dynamic pricing models for HSR in the aviation competition environment considering passenger cancellation, no-shows and overbooking. However, they did not optimize the overbooking strategy. Deng et al. [27] developed an overbooking model for a single train and a single OD in HSR and employed a genetic algorithm to solve the mathematical model and analyze overbooking rules. Furthermore, comprehensive research that integrates overbooking with seat allocation optimization in the railway remains scarce, and the aforementioned studies on seat allocation did not incorporate overbooking. In practical implementation, the application of overbooking in the railway faces many challenges, such as the identification of DB passengers. Drawing upon practice experience in aviation, the railway operator can inform passengers of the potential DB risk and determine DB passengers according to the “first-come, first-served” principle. On the other hand, the railway operator can offer upgraded seating or arrange alternative trains for DB passengers to accommodate DB passengers’ travel demands.

In conclusion, the research and practical implementation of overbooking in the railway is presently relatively limited, necessitating further quantitative investigations and exploration of comprehensive optimization methods to facilitate more efficient operations and higher utilization of seating resources. Given the above-mentioned research gaps, considering passenger demand and passenger no-show behavior uncertainty, this paper investigates the joint optimization of overbooking and seat allocation for HSR. Aiming to maximize railway expected revenue, we establish a non-linear stochastic programming model considering train capacity, demand, and denied boarding constraints. Then, a multi-level iterative optimization method is designed to solve the seat allocation scheme. Finally, numerical studies are tested to evaluate the effectiveness of the proposed model and solution algorithm.

The rest of this paper is structured as follows. Section 2 presents related assumptions and the integrated optimization model for overbooking and seat allocation under passenger no-show and demand uncertainty. Section 3 describes the designed multi-level iterative optimization approach, and numerical studies conducted to evaluate the performance of the model and solution algorithm are presented in Section 4. Finally, Section 5 summarizes our conclusions and directions for future research.

## 2 Model formulation

### 2.1 Assumptions and notations

To develop the mathematical optimization model, this study makes the following assumptions:

1. The demands between each OD are independent and stochastic, following a certain distribution.
2. Only a single-seat class is considered. The overbooking and seat allocation problem for different seat classes can be independently optimized.
3. After purchasing tickets, passengers can only be in one of two states: show or no-show.
4. The refund fee rate for no-show passengers is constant and independent of the time of ticket cancellation.
5. The resale of refunded tickets is not taken into account.

In addition, the major notations used in the modeling formulation are summarized in Table 1.

**Table 1:** Major notations.

Notations	Definitions
Indices	
*S*	The number of stations
*i*,*j*	station
(*i*,*j*)	OD from the origin station *i* to destination station *j*
*h*	High-speed train
*l*	Leg, i.e., the section between two adjacent stop stations
〈(*i*,*j*)|*h*〉	Product, i.e., the transportation service for OD (*i*,*j*) served by train *h*
Sets	
*W*	The set of ODs
*H*	The set of trains
*H*_*ij*_	The set of trains serving OD (*i*,*j*)
Parameters	
*C*_*h*_	The seating capacity for train *h*
*S*_*h*_	The number of stop stations
$f_{ij}^{h}$	The price of product 〈(*i*,*j*)|*h*〉
*γ*	The refund fee rate
*α*	A certain multiple of the ticket price
*β*	The maximum overbooking rate
*τ*_0_	The maximum DB rate
Variables	
$m_{ij}^{h}$	The number of tickets allocated to product 〈(*i*,*j*)|*h*〉
$r_{ij}^{h}$	The number of DB passengers for product 〈(*i*,*j*)|*h*〉
*q*_*ij*_	The passenger demand for OD (*i*,*j*)
$n_{ij}^{h}$	The number of no-show passengers for product 〈(*i*,*j*)|*h*〉
$\delta_{ij}^{hl}$	A binary variable denotes the relationship between product 〈(*i*,*j*)|*h*〉 and leg *l*
*R*_*r*_	The railway ticket revenue
*R*_*c*_	The railway costs

### 2.2 Problem description

This paper takes one direction of an HSR with *S* station as the research object. OD (*i*,*j*) denotes the origin station *i* and destination station *j* of passenger travel, 1≤*i*≤*j*≤*S*.*W* represents the set of ODs. *h* denotes a high-speed train running on the HSR line and *H* denotes the set of trains. *H*_*ij*_ denotes the set of trains serving OD (*i*,*j*). For train *h*, the seating capacity is denoted as *C*_*h*_ and the number of stop stations is denoted as *S*_*h*_. The section between two adjacent stop stations is denoted as leg *l*. Product 〈(*i*,*j*)|*h*〉 denotes the transportation service for OD (*i*,*j*) served by train *h*. $f_{ij}^{h}$ denotes the price of product 〈(*i*,*j*)|*h*〉, which is a known variable. $m_{ij}^{h}$ denotes the number of tickets allocated to product 〈(*i*,*j*)|*h*〉, which is a decision variable.

The passenger demand for OD (*i*,*j*), denoted as *q*_*ij*_, is stochastic and follows a specific distribution function. Similar to the existing studies such as Wang et al. [28] and Yan et al. [5], the demand *q*_*ij*_ in this study can be assumed to follow a Poisson distribution. It is noted that the proposed model and solution method in this paper is also applicable to other forms of demand distribution.

The seat allocation scheme is typically determined based on the passenger demand. Thus, $m_{ij}^{h}$ is subject to the stochastic nature of *q*_*ij*_. After purchasing tickets, passengers may refund tickets or directly give up boarding the train due to personal itinerary changes, bad weather or traffic delays, which is regarded as passenger no-show behavior. Passenger no-show behavior can lead to the wastage of seat value. However, an overbooking strategy can make up for the empty seat loss caused by no-show behavior to some extent. HSR overbooking refers to selling more tickets to passengers than the train seating capacity, that is, the number of tickets that can be sold for each leg is increased from the train seating capacity to the sum of the train seating capacity and the number of overbookings.

$n_{ij}^{h}$ denotes the number of no-show passengers for product 〈(*i*,*j*)|*h*〉, which is a random variable and follows a certain distribution. No-show passengers can request ticket refunds from the railway operators which will refund the ticket price and charge a certain refund fee. The refund fee is equal to the ticket price multiplied by the refund fee rate *γ*.

For leg *l* of train *h*, if the number of boarding passengers exceeds the train seating capacity, i.e., $\sum_{(i,j)\in W}\delta_{ij}^{hl}(m_{ij}^{h}-n_{ij}^{h})>C_{h}$, it is necessary to reject passengers beyond the seating capacity. $\delta_{ij}^{hl}$ is a binary variable, which equals one if product 〈(*i*,*j*)|*h*〉 covers leg *l* and zero otherwise. The railway operator needs to determine the number of DB passengers $r_{ij}^{h}$ for each product and provide compensation to the DB passengers. This study assumes that the unit compensation cost for each passenger is a certain multiple *α* of the ticket price. If the number of boarding passengers is less than the train seating capacity, i.e., $\sum_{(i,j)\in W}\delta_{ij}^{hl}(m_{ij}^{h}-n_{ij}^{h})\leq C_{h}$, the number of DB passengers for the product is zero.

For railway operators, passenger no-show behavior results in refund cost, while DB behavior leads to DB cost, which has a negative impact on maximizing railway revenue. Considering the randomness of passenger demand and no-show behavior, it is necessary to determine a reasonable seat allocation scheme for each product to minimize the railway cost and maximize the railway expected revenue.

### 2.3 Mathematical model

#### 2.3.1 Objective function

Due to the randomness of passenger demand and no-show behavior, the optimization objective of this study is to maximize the railway expected revenue. For given realizations of the number of no-show passengers and demand, the railway revenue consists of two components: railway ticket revenue *R*_*r*_ and railway costs *R*_*c*_.

The railway ticket revenue *R*_*r*_ is calculated as the sum of the ticket price of each product multiplied by the number of allocated tickets, as shown below:

$$R_{r}=\sum_{h\in H_{ij}}\sum_{(i,j)\in W}f_{ij}^{h}m_{ij}^{h}$$ (1)

The railway costs *R*_*c*_ include refund cost and DB cost, which is given below. The refund cost is incurred due to ticket refunds by no-show passengers and the DB cost arises from the compensation provided by the railway operators to DB passengers.


$$R_{c}=\sum_{h\in H_{ij}}\sum_{(i,j)\in W}(1-\gamma )f_{ij}^{h}n_{ij}^{h}+\sum_{h\in H_{ij}}\sum_{(i,j)\in W}\alpha f_{ij}^{h}r_{ij}^{h}$$ (2)

Affected by the demand uncertainty, the railway operators allocate tickets to each product, resulting in railway ticket revenue. Then, the railway costs may occur influenced by random passenger no-show behavior. Therefore, the objective function can be expressed as follows:

$$\max\;R=E(R_{r}-E(R_{c}))$$ (3)

#### 2.3.2 Constraints

This study takes various constraints into account, such as train capacity constraints, demand constraints, train seating capacity constraints after DB, DB rate constraints and variable range constraints.

(1) Train capacity constraints. For leg *l* of train *h*, the total allocated tickets cannot exceed the maximum transportation capacity. The seating capacity for each leg of the same train is equal. Considering the overbooking strategy, the maximum transportation capacity of leg *l* is the sum of the seating capacity and the allowed overbooking capacity. The allowed overbooking capacity is a certain proportion of the train seating capacity.

$$\sum_{(i,j)\in W}\delta_{ij}^{hl}m_{ij}^{h}\leq C_{h}(1+\beta ),h\in H,1\leq l\leq S_{h}-1$$ (4)

where $\delta_{ij}^{hl}$ denotes the relationship between product 〈(*i*,*j*)|*h*〉 and leg *l*. If product 〈(*i*,*j*)|*h*〉 covers leg *l*, $\delta_{ij}^{hl}=1$; otherwise, $\delta_{ij}^{hl}=0$. The maximum overbooking rate *β* represents the ratio of the allowed overbooking capacity to train seating capacity.

(2) Demand constraints. The total allocated tickets for OD (*i*,*j*) cannot exceed the demand of this OD. The demand *q*_*ij*_ of OD (*i*,*j*) is stochastic.

$$\sum_{h\in H_{ij}}m_{ij}^{h}\leq q_{ij},(i,j)\in W$$ (5)

(3) Train seating capacity constraints after DB. Due to the implementation of an overbooking strategy, the total allocated tickets for leg *l* of train *h* may exceed the train seating capacity. When the realization of the number of no-show passengers is known, DB may occur if the number of boarding passengers exceeds the train seating capacity. Thus, the number of actual boarding passengers for leg *l* of train *h* cannot exceed the train seating capacity after DB.

$$\sum_{(i,j)\in W}\delta_{ij}^{hl}(m_{ij}^{h}-n_{ij}^{h}-r_{ij}^{h})\leq C_{h},h\in H,1\leq l\leq S_{h}-1$$ (6)

(4) DB rate constraints. To avoid the negative impact on the railway’s reputation and the passenger travel inconvenience due to excessive DB, it is necessary to limit the number of DB passengers for each product to a small proportion. *τ*_0_ represents the maximum DB rate, which is set by the railway operators or government authorities.

$$r_{ij}^{h}/m_{ij}^{h}\leq \tau_{0},(i,j)\in W,h\in H_{ij}$$ (7)

(5) Variable range constraints. The number of tickets allocated to each product and the number of DB passengers are non-negative integers. The number of DB passengers for each product cannot exceed the number of allocated tickets.

$$m_{ij}^{h}\in N^{+},(i,j)\in W,h\in H_{ij}$$ (8)

$$r_{ij}^{h}\in N^{+},(i,j)\in W,h\in H_{ij}$$ (9)

$$r_{ij}^{h}\leq m_{ij}^{h},(i,j)\in W,h\in H_{ij}$$ (10)

In all, considering the randomness of passenger demand and no-show behaviors, the proposed joint optimization model of overbooking and seat allocation for HSR is a non-linear stochastic integer programming (SOM) with the aim of maximizing railway expected revenue, which is represented by Eqs (3)–(10). Eq (3) represents the objective function, which is non-linear. Eqs (4), (5) and (8) are ticket-related constraints, which depend on the realization of demand. Eqs (6), (7), (9) and (10) are DB-related constraints, which depend on the realization of passenger no-show behaviors. All constraints are linear constraints. The decision variables include the number of tickets allocated to each product $m_{ij}^{h}$ and the number of DB passengers for each product $r_{ij}^{h}$.

## 3 Solution method

### 3.1 A framework of joint optimization of overbooking and seat allocation for high-speed trains

**Fig 1:**
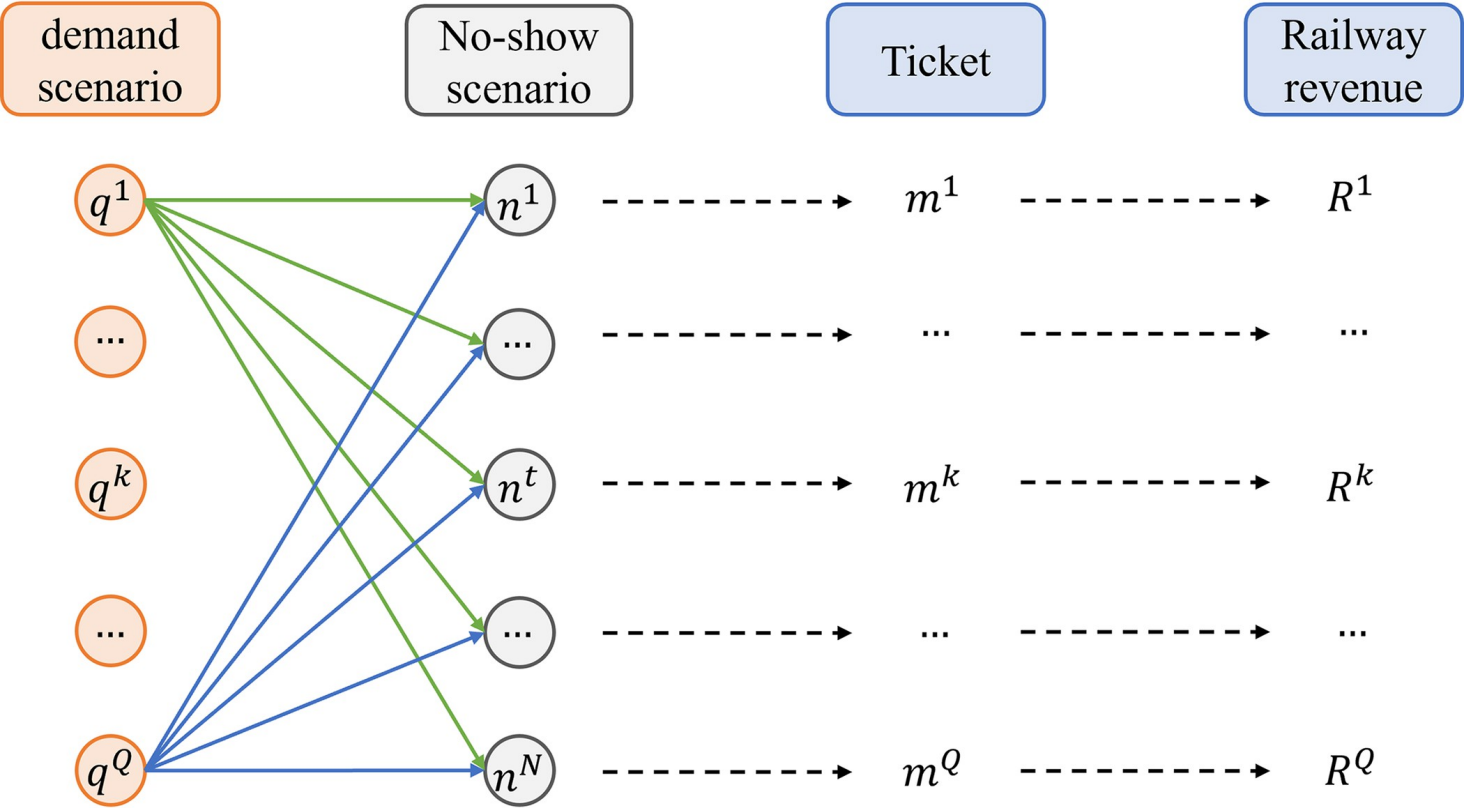
Relationship between demand scenario and no-show scenario.

The joint optimization model of overbooking and seat allocation for high-speed trains are influenced by the randomness of the number of no-show passengers $n_{ij}^{h}$ and passenger demand *q*_*ij*_, making it difficult to directly solve it. The realization of passenger demand and subsequent passenger no-show behavior follows a sequential order. The seat allocation scheme for each product is optimized based on the passenger demand during the booking horizon. Before the train departs, passenger no-show behavior occurs; then, whether or not to DB is determined. One realization of the passenger demand is linked with multiple realizations of passenger no-show. To tackle this stochastic programming model, this paper adopts the sampling average approximation method, in which the random variables are represented by samples and the stochastic programming is transformed into a deterministic programming to be solved. In the SOM model, the samples of the random variable *q*_*ij*_ is denoted by $q_{ij}^{1},q_{ij}^{2},\ldots ,q_{ij}^{Q}$, and the samples of the random variable $n_{ij}^{h}$ is denoted by $n_{ij}^{h1},n_{ij}^{h2},\ldots ,n_{ij}^{hN}$. Each sample is regarded as a scenario, for example, $q_{ij}^{1}$ denotes the first demand scenario and $n_{ij}^{h1}$ denotes the first passenger no-show scenario. One demand scenario is one realization of passenger demand for all ODs and one passenger no-show scenario is one realization of the number of passenger no-shows for all ODs. Multiple demand scenarios are multiple realizations (i.e., multiple simulations) of passenger demand for all ODs. Different demand scenarios are independent with each other and they can be randomly generated independently. Different passenger no-show scenarios are similar. The relationship between the demand scenario and passenger no-show scenario is illustrated in Fig 1, where, ***n***^*t*^ denotes passenger no-show scenario *t*, and ***q***^*k*^ represents demand scenario *k*. The occurrence of no-show scenarios is dependent on the realization of each demand scenario, and the railway revenue associated with the demand scenario ***q***^*k*^ is denoted as *R*^*k*^. Consequently, under scenario ***q***^*k*^, the seat allocation scheme is denoted as ***m***^*k*^.

To reduce the solution difficulty and complexity, this study decomposes the optimization problem into *Q* sub-problems based on demand scenarios. Each demand scenario ***q***^*k*^ corresponds to sub-problem *k*. The optimization result for each sub-problem *k* is represented as ***m***^*k*^. Investigating the effectiveness of seat allocation scheme ***m***^*k*^ across different demand scenarios and calculating the average revenue generated in other demand scenarios by seat allocation scheme ***m***^*k*^, the seat allocation scheme with the highest average revenue is selected as the final solution.

Based on the aforementioned analysis, this study generates demand scenarios and passenger no-show scenarios according to the sampling average approximation method and decomposes the optimization problem into the joint optimization of overbooking and seat allocation under a single demand scenario (Algorithm 2), and the seat allocation adjustment under other demand scenarios (Algorithm 3). The former can be further decomposed into two sub-problems to be iteratively solved according to the randomness of passenger no-shows. The latter involves adjusting the seat allocation scheme based on the seating capacity after DB in other demand scenarios and calculating the corresponding revenue.

The solution steps of the joint optimization model of overbooking and seat allocation for HSR are given below:

Algorithm 1. Solution of the joint optimization of overbooking and seat allocation for HSR.

Step 1: Initialize. Generate *Q* demand scenarios and *N* no-show scenarios.

Step 2: Set *k* = 1.

Step 3: Solve the best seat allocation scheme ***m***^*k*^ and the best railway revenue *R*^*k*^ under the *k*^*th*^ demand scenario and *N* no-show scenarios (Algorithm 2).

Step 4: Set *g* = 1.*G* denotes the set of demand scenarios that do not contain scenario *k*.

Step 5: Solve the railway revenue $R_{g}^{k}$ for the seat allocation scheme ***m***^*k*^ of demand scenario *k* under the *g*^*th*^ demand scenario and *N* no-show scenarios (Algorithm 3).

Step 6: If *g*<|*G*|,*g* = *g*+1, go to Step 5; otherwise, go to Step 7.

Step 7: The railway expected revenue under all demand scenarios for the seat allocation scheme ***m***^*k*^ of demand scenario *k* is $R_{a}^{k}=\frac{R^{k}+\sum_{g=1}^{\left|G\right|}R_{g}^{k}}{Q}$.

Step 8: If *k*<*Q*,*k* = *k*+1, go to Step 3; otherwise, go to Step 9.

Step 9: Select the seat allocation scheme for the demand scenario with the largest expected revenue (i.e., $\max\left\{R_{a}^{k}|k=1,2,\ldots ,Q\right\}$) as the final solution.

In Algorithm 1, Step 3 optimizes the seat allocation under a given demand scenario considering the randomness of passenger no-show behavior, which is solved by Algorithm 2. Step 5 aims to calculate the revenue under different demand scenarios for the seat allocation scheme obtained by Step 3 and adjust the seat allocation according to whether the seat allocation scheme satisfies the constraints, which is solved by Algorithm 3.

### 3.2 Joint optimization of overbooking and seat allocation under a given demand scenario

In the SOM model, the objective function in Eq (3) consists of three terms. The first term is related to the demand, while the second and third terms are associated with passenger no-show behavior. The constraints in Eqs (4), (5) and (8) are linked to the realization of demand and the number of tickets allocated to each product $m_{ij}^{h}$. The constraints in Eqs (6), (7), (9) and (10) are related to the realization of passenger no-show behavior and the number of DB passengers for each product $r_{ij}^{h}$. Thus, the joint optimization of overbooking and seat allocation problem under a given demand scenario *k* and multiple no-show scenarios can be decomposed into two sub-problems for iteratively solving based on the model characteristics. The sub-problem 1 considers the objective function and constraints related to demand and optimizes the number of tickets allocated to each product $m_{ij}^{h}$. Based on the optimized results obtained by sub-problem 1, sub-problem 2 aims to minimize the railway cost caused by passenger no-show behaviors considering multiple passenger no-show scenarios.

For sub-problem 1, if the current seat allocation scheme does not satisfy the train seating capacity constraint after DB, a penalty term is introduced in the objective function to reduce the number of tickets with the aim of maximizing railway revenue. The objective function for sub-problem 1 is as follows:

$$\max R_{upper}=\sum_{h\in H_{ij}}\sum_{(i,j)\in W}f_{ij}^{h}m_{ij}^{h}-\sum_{h\in H_{ij}}\sum_{(i,j)\in W}\rho_{1}\theta_{ij}^{h}f_{ij}^{h}m_{ij}^{h}$$ (11)

The constraints are Eqs (4), (5) and (8). $\theta_{ij}^{h}$ represents whether the leg related to product 〈(*i*,*j*)|*h*〉 satisfies the train seating capacity constraint after DB. If it satisfies the train seating capacity constraint after DB, $\theta_{ij}^{h}=0$; otherwise, $\theta_{ij}^{h}=1$. The initial value of $\theta_{ij}^{h}$ is a zero vector. *ρ*_1_ denotes the penalty coefficient, where $\rho_{1}\in [\underline{\rho },{\bar{\rho }}_{1}]$ and it can be gradually increased with a step of 10.

After obtaining the seat allocation scheme $m_{ij}^{h}$, the maximum number of DB passengers $r_{0ij}^{h}$ corresponding to the maximum DB rate can be calculated, as shown in Eq (12). Taking $m_{ij}^{h}$ and $r_{0ij}^{h}$ as input, whether each passenger no-show scenario satisfies the train seating capacity constraint after DB, as defined in Eq (6).


$$r_{0ij}^{h}=\tau_{0}m_{ij}^{h}$$ (12)

If all passenger no-show scenarios satisfy the train seating capacity constraint after DB, sub-problem 2 is solved. Sub-problem 2 includes the objective function Eq (13) and constraints Eqs (6), (7), (9) and (10) that are related to passenger no-show behavior.


$$\min\;R_{lower}=\frac{1}{N}\sum_{t=1}^{N}R_{c}(n^{t},m_{ij}^{h})$$ (13)

To facilitate solving sub-problem 2, a penalty term *ρ*_2_ is introduced to incorporate Eq (7) as part of the objective function Eq (13). Thus, in sub-problem 2, the objective function is as follows, and the constraints are Eqs (6), (9) and (10).


$$\min\;R_{lower}=\frac{1}{N}\sum_{t=1}^{N}\left(R_{c}(n^{t},m_{ij}^{h})-\rho_{2}\max\left\{\frac{r_{ij}^{h}}{m_{ij}^{h}}-\tau_{0},0\right\}\right)$$ (14)

After optimizing, the best railway costs of sub-problem 2 is *R*_lower_*. Thus, for demand scenario *k*, the optimized seat allocation scheme is $m_{ij}^{h}$ and the railway revenue *R*^*k*^ can be calculated by Eq (15).


$$R^{k}=\sum_{h\in H_{ij}}\sum_{(i,j)\in W}f_{ij}^{h}m_{ij}^{h}-{R_{lower}}^{*}$$ (15)

If there exist passenger no-show scenarios that do not satisfy the train seating capacity constraint after DB, the seat allocation scheme obtained from sub-problem 1 needs to be adjusted. If the legs related to product 〈(*i*,*j*)|*h*〉 do not meet Eq (6), $\theta_{ij}^{h}=1$. Substitute the new value of $\theta_{ij}^{h}$ into sub-problem 1 and continue solving it. Until the seat allocation scheme $m_{ij}^{h}$ for all scenarios satisfies all constraints, the optimal ticket scheme is obtained.

**Fig 2:**
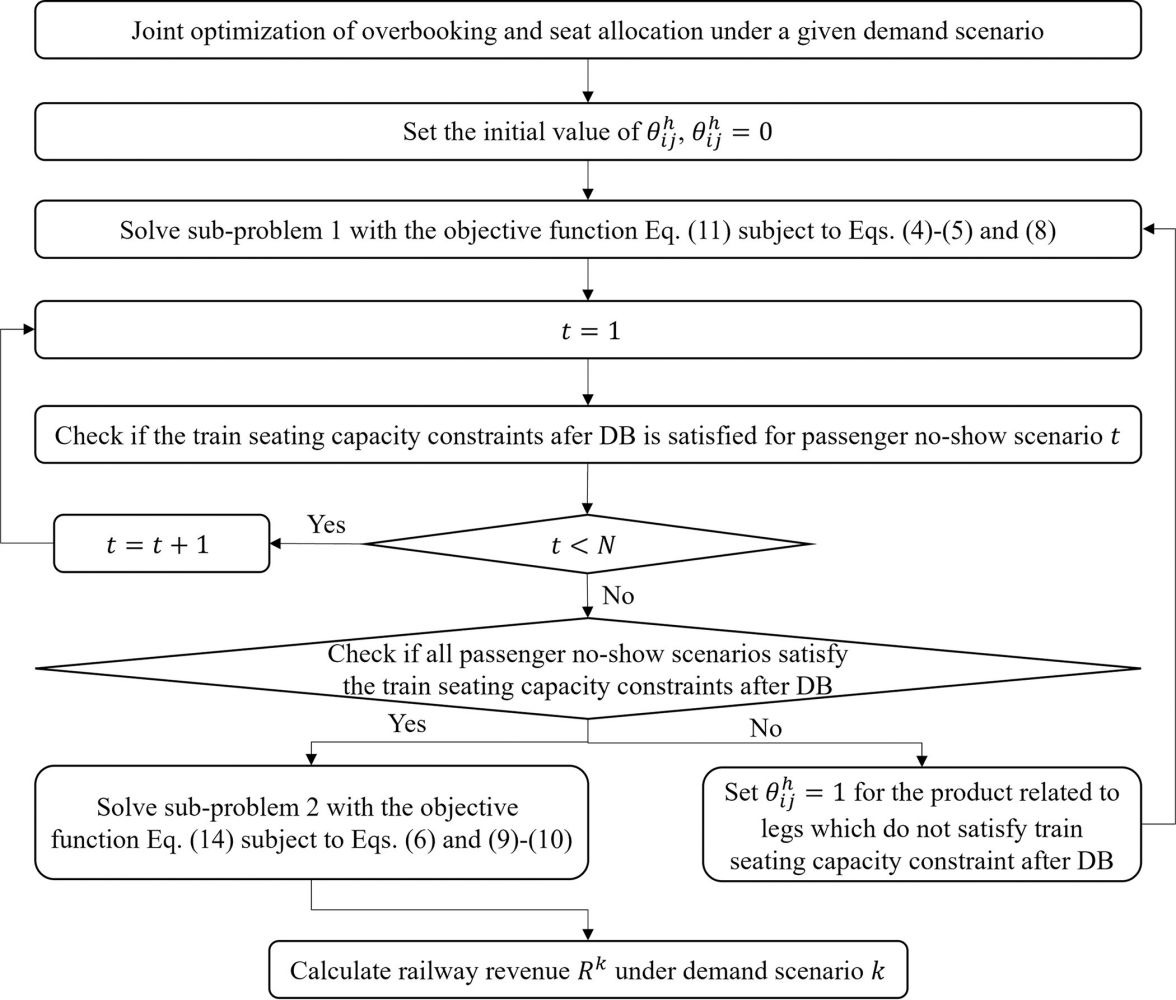
The flowchart of Algorithm 2.

The solution steps of the seat allocation under a given demand scenario considering the randomness of passenger no-show behavior (Algorithm 2) are given below. The flowchart of Algorithm 2 is presented in Fig 2. The relationship between subproblem 1 and subproblem 2 is: Sub-problem 1 provides seat allocation scheme $m_{ij}^{h}$ for sub-problem 2 and sub-problem 2 is used to solve the railway cost under multiple passenger no-show scenarios under the seat allocation scheme obtained from sub-problem 1.

Algorithm 2. Seat allocation under a given demand scenario considering no-show behavior.

Step 1: Initialization demand scenario *k* and *T* passenger no-show scenarios. Set $\theta_{ij}^{h}=0$.

Step 2: Solve sub-problem 1 and obtain the seat allocation scheme $r_{0ij}^{h}$. Calculate the maximum number of DB passengers ${r_{0}}_{ij}^{h}$ by Eq (12).

Step 3: Check whether it satisfies the train seating capacity constraints after DB for each passenger no-show scenario. For product 〈(*i*,*j*)|*h*〉 related to legs that do not satisfy the train seating capacity constraint after DB, set $\theta_{ij}^{h}=1$; otherwise, $\theta_{ij}^{h}=0$.

Step 4: If $\theta_{ij}^{h}$ is a zero vector, solve sub-problem 2 and obtain the railway costs *R*_lower_*. Calculate the railway revenue *R*^*k*^ under demand scenario *k* according to Eq (15), go to Step 5. Otherwise, take $\theta_{ij}^{h}$ as the input of sub-problem 1 and go to Step 2.

Step 5: Stop the algorithm and output the seat allocation scheme $m_{ij}^{h}$ and railway revenue *R*^*k*^ under demand scenario *k*.

### 3.3 Revenue calculation for the seat allocation scheme under different demand scenarios

Sub-problem *k* optimizes the seat allocation scheme under the given demand scenario *k*. The revenue $R_{g}^{k}$ under demand scenario *g* can be calculated based on the best seat allocation scheme ***m***^*k*^ under demand scenario *k*. Affected by the stochasticity of the demand, the seat allocation scheme ***m***^*k*^ may not satisfy all the constraints under the demand scenario *g*, such as the demand constraints. If the demand constraints are satisfied, the revenue $R_{g}^{k}$ is calculated directly. Otherwise, the seat allocation scheme needs to be adjusted to obtain a feasible solution under the demand scenario *g*, and then the revenue $R_{g}^{k}$ is calculated.

The relationship between sub-problem *k* and Algorithm 3 is: sub-problem *k* provides inputs for Algorithm 3 and it is the calculation basis for Algorithm 3. Algorithm 3 can be called by Algorithm 1 to provide the basis for calculating the expected railway revenue under all demand scenarios for the optimized seat allocation scheme of sub-problem *k*.

The solution steps of the revenue calculation for the seat allocation scheme under different demand scenarios (Algorithm 3) are given below:

Algorithm 3. Revenue calculation for the seat allocation scheme under different demand scenarios.

Step 1: Initialize. Set *u* = 0, and ***m***^*k*^_*u*_ = ***m***^*k*^.

Step 2: Calculate the revenue *R*_*temp*_ caused by the seat allocation scheme ***m***^*k*^_*u*_ and the number of DB passengers ${r_{g}^{k}}_{u}$, which can be solved by the mathematical model with the objective function Eq (16), subject to Eqs (6), (9) and (10).

$$\max\;R_{temp}=\sum_{h\in H_{ij}}\sum_{(i,j)\in W}f_{ij}^{h}m_{ij}^{h}-\frac{1}{N}\sum_{t=1}^{N}\left(R_{c}(n^{t},m_{ij}^{h})-\rho_{2}\max\left\{\frac{r_{ij}^{h}}{m_{ij}^{h}}-\tau_{0},0\right\}\right)$$ (16)

Step 3: If the seat allocation scheme ***m***^*k*^_*u*_ satisfied the demand constraints Eq (5) under demand scenario *g*, the revenue under demand scenario *g* is $R_{g}^{k}=R_{temp}$, go to Step 7. Otherwise, *u* = *u*+1, go to Step 4.

Step 4: If any of the following criteria are satisfied: (1) the number of iterations *u* exceeds the maximum number of iterations *U*; or (2) the revenue does not change for several consecutive iterations, then the revenue under demand scenario *g* is $R_{g}^{k}=R_{temp}$ and go to Step 7. Otherwise, go to Step 5.

Step 5: Calculate the maximum value of the actual DB rate for each product in each no-show scenario, $\tau_{real}=\max\frac{{r_{g}^{k}}_{u}}{{m^{k}}_{u}},\tau_{real}$ is a vector containing the maximum actual DB rate $\tau_{ij}^{h}$ for each product.

Step 6: Calculate the new seat allocation scheme ***m***^*k*^_*u*_ according to the maximum actual DB rate by solving the mathematical model with objective function Eq (17) subject to Eqs (4)–(6) and (8)-(10). *ρ*_3_ denotes the penalty for products that do not satisfy the DB rate constraints, where $\rho_{3}\in [{\underline{\rho }}_{3},{\bar{\rho }}_{3}]$ and it gradually increases with a step of 10. Go to Step 2.

$$\max\;R_{temp2}=R_{temp}-\sum_{h\in H_{ij}}\sum_{(i,j)\in W}\rho_{3}\tau_{ij}^{h}f_{ij}^{h}m_{ij}^{h}$$ (17)

Step 7: Output the revenue $R_{g}^{k}$ under demand scenario *g* for the seat allocation scheme obtained in demand scenario *k*.

## 4 Numerical studies

**Fig 3:**
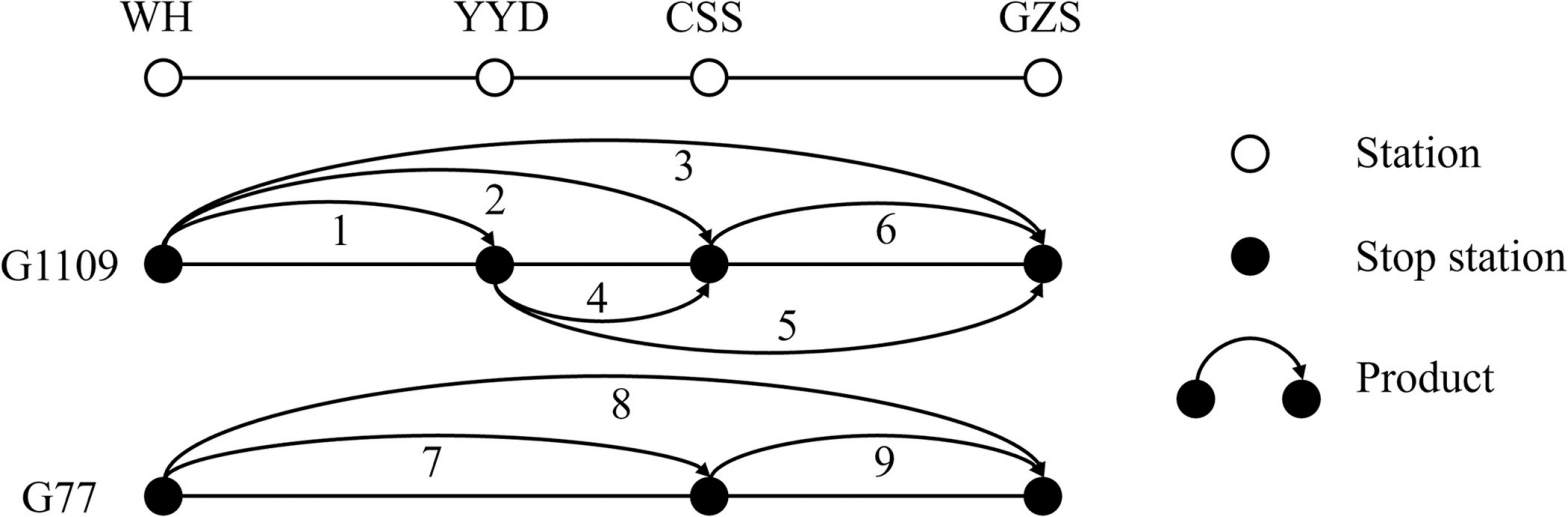
Train stopping plan.

This section takes two trains G1109 and G77 on the Wuhan-Guangzhou HSR line as the research object and solves the seat allocation scheme of this numerical instance to verify the effectiveness of the proposed model and algorithm. Then, sensitivity analysis is carried out on some crucial parameters such as maximum overbooking rate and maximum DB rate to explore the influence of these parameters on the optimization results. The train stopping plan for this numerical example is shown in Fig 3. There are 9 products in total. The price and initial number of tickets for each product are shown in Table 2.

**Table 2:** Price and the initial number of tickets for each product.

Product number	OD	Train	Price	The initial number of tickets
1	WH-YYD	G1109	96	150
2	WH-CSS	G1109	162	150
3	WH-GZS	G1109	374	238
4	YYD-CSS	G1109	66	50
5	YYD-GZS	G1109	341	100
6	CSS-GZS	G1109	282	200
7	WH-CSS	G77	162	300
8	WH-GZS	G77	374	238
9	CSS-GZS	G77	282	300

The number of no-show passengers for product 〈(*i*,*j*)|*h*〉 is a random variable following a Poisson distribution, which is equal to the number of allocated tickets multiplied by the passenger no-show rate. This paper generates the passenger no-show rate following a Poisson distribution to express the randomness of the number of no-show passengers. The mean value of the passenger no-show rate is set as *p* = 0.1. The maximum overbooking rate is *β* = 0.1. The maximum DB rate is *τ*_0_ = 0.05. The passenger refund fee rate is *γ* = 0.1. The DB compensation multiplier is *α* = 2. The passenger time value is ν = 0.5 yuan/min. The seating capacity of the two trains is *C*_*h*_ = 538. Assuming that the passenger demand follows a Poisson distribution as literature (Yan et al. [5] and Wang et al. [28]), the demand intensity parameters of Poisson distribution for each OD is shown in Table 3. One demand scenario represents one realization of passenger demand for all ODs. During the optimization process, the demand values for one demand scenario following a Poisson distribution with the demand intensity parameters given in Table 3 are randomly generated. This process is repeated to randomly generate multiple demand scenarios.

**Table 3:** Demand intensity parameters of Poisson distribution for each OD.

OD number	OD	Demand intensity parameter
1	WH-YYD	250
2	WH-CSS	660
3	WH-GZS	650
4	YYD-CSS	500
5	YYD-GZS	200
6	CSS-GZS	700

### 4.1 Optimized results

Passenger demand and passenger no-show behavior have randomness, this paper simulates passenger demand scenarios and passenger no-show scenarios based on the sampling averaging approximation method. The number of scenarios on the one hand affects the computational complexity, and on the other hand, also affects the solution effect. To facilitate the analysis, it is assumed that the number of demand scenarios and the number of passenger no-show scenarios are the same. This section analyzes the optimal railway expected revenue, revenue increase rate and computation time under different numbers of scenarios, as shown in Table 4. With the increase in the number of demand scenarios and passenger no-show scenarios, the railway expected revenue is in a fluctuating state due to the randomness of demand and passenger no-show behavior, but the calculation time rises significantly. When the number of demand scenarios and the number of passenger no-show scenarios is 50, the railway expected revenue is 439958.24 yuan, and the calculation time is up to 14.5 hours. Compared to the case of 50 demand scenarios and no-show scenarios, when the number of demand scenarios and no-show scenarios is 10, the gap between the railway expected revenues is the smallest, with a difference of only 0.06%, while the computation time is significantly shortened, which saves about 98% of the time. Therefore, in the subsequent research, the number of scenarios is set to 10 to facilitate a better solution in an acceptably shorter computation time.

**Table 4:** The railway expected revenue, revenue increase rate and calculation time under different numbers of scenarios.

Number of demand scenarios	Number of no-show scenarios	Railway expected revenue (Yuan)	Initial revenue (Yuan)	Revenue increase rate	Calculation time (s)
1	1	446012.86	415403.99	0.0737	1.63
2	2	439816.43	411711.47	0.0683	3.84
3	3	439325.79	405594.91	0.0832	13.20
4	4	443273.49	405812.66	0.0923	21.70
5	5	444235.28	406142.52	0.0938	39.66
6	6	442703.23	405812.06	0.0909	72.79
7	7	434384.80	406012.79	0.0699	153.80
8	8	444167.54	405411.71	0.0956	324.38
9	9	445302.98	405758.64	0.0975	544.67
10	10	440203.63	406071.24	0.0841	1006.80
15	15	436243.85	406353.47	0.0736	1827.75
20	20	441936.26	37857.59	0.0889	5929.30
25	25	437143.16	405243.87	0.0787	9714.24
30	30	434962.12	38942.43	0.0746	17016.09
50	50	439958.24	39877.75	0.0894	52110.21

Taking 10 demand scenarios and no-show scenarios as an example, the seat allocation scheme for each product after optimization is shown in Table 5. ODs WH-CSS, WH-GZS and CSS-GZS are both served by trains G1109 and G77. Since G1109 also serves YYD station and needs to allocate tickets for YYD station, and the sum of prices of OD WH-YYD and OD YYD-CSS is higher than that of OD WH-CSS, the passenger demand of OD WH-CSS is mainly transported by G77. Correspondingly, the passenger demand of OD CSS-GZS is also preferentially satisfied by G77. From Table 5, it can be seen that the number of tickets for each OD is less than the demand, and the number of tickets for each leg is less than the maximum transportation capacity, indicating that the solution results satisfy all the constraints considered in the optimization model. For instance, taking train G1109 as an example, the number of tickets for leg WH-YYD is equal to the sum of the number of tickets for ODs WH-YYD, WH-CSS and WH-GZS, i.e., 246+335+10 = 591, which is less than the maximum transportation capacity.

**Table 5:** The seat allocation scheme for each product after optimization.

Product number	OD	Train	Number of tickets
1	WH-YYD	G1109	246
2	WH-CSS	G1109	335
3	WH-GZS	G1109	10
4	YYD-CSS	G1109	54
5	YYD-GZS	G1109	192
6	CSS-GZS	G1109	389
7	WH-CSS	G77	290
8	WH-GZS	G77	301
9	CSS-GZS	G77	290

### 4.2 Differentiated price versus uniform price

In Section 4.1, the trains serving the same OD adopt the same price level, so that the number of tickets is allocated based on demand only. This section explores the effect of differentiated prices for parallel trains on the seat allocation results. For ODs WH-CSS, WH-GZS, and CSS-GZS, the price for G1109 is 1.2 times the original price and the price for G77 is 0.8 times the original price. The differentiated prices and optimized prices for each product are shown in Table 6. It can be seen that after the price increase of G1109, the seat allocation is constrained by the optimization objective of maximizing railway expected revenue and the demands of ODs WH-CSS and CSS-GZS are met by G1109 as much as possible. The numbers of tickets of ODs WH-CSS and CSS-GZS served by G77 are significantly reduced, and G77 mainly meets the demand of OD WH-GZS. The final revenue for the differentiated price case is 439531.76 yuan, an increase in revenue of approximately 11.08%.

**Table 6:** The number of tickets after optimization for differentiated price and uniform price cases.

Number	OD	train	Price (U)	Number of tickets (U)	Price (D)	Number of tickets (D)
1	WH-YYD	G1109	96	246	96	45
2	WH-CSS	G1109	162	335	194	536
3	WH-GZS	G1109	374	10	448	10
4	YYD-CSS	G1109	66	54	66	35
5	YYD-GZS	G1109	341	192	341	10
6	CSS-GZS	G1109	282	389	338	571
7	WH-CSS	G77	162	290	129	105
8	WH-GZS	G77	374	301	299	486
9	CSS-GZS	G77	282	290	225	105

Note: U denotes the uniform price, and D denotes the differentiated price.

### 4.3 Sensitivity analysis

This section conducts a sensitivity analysis to explore the impact of important parameters such as passenger no-show rate, maximum overbooking rate, maximum DB rate, and DB compensation multiplier on railway expected revenue. The price, initial number of tickets, and other parameter settings for each product are the same as in Section 4.1.

(1) Passenger no-show rate

According to the data statistics, the average passenger no-show rate in airlines in China is 5%-15%. Therefore, let the passenger no-show rate varies from 0.05 to 0.2 with a step of 0.05, the railway expected revenue at different levels of passenger no-show rate is shown in Table 7. Assuming that the maximum overbooking rate and the maximum DB rate are 0.1 and 0.05, respectively, when the passenger no-show rate is 0.05, excessive overbooking is likely to cause a decrease in the railway expected revenue; when the passenger no-show rate is 0.1, the railway expected revenue is the highest; and the railway expected revenue gradually decreases when the passenger no-show rate is further increased. Passenger no-show rate reflects the randomness of passenger no-show, the larger the passenger no-show rate, the greater the empty seat loss.

**Table 7:** Changes in railway expected revenue with passenger no-show rate.

Passenger no-show rate	Railway expected revenue (Yuan)
0.05	372949.10
0.1	439223.33
0.15	436013.80
0.2	413712.12

(2) Maximum overbooking rate

**Fig 4:**
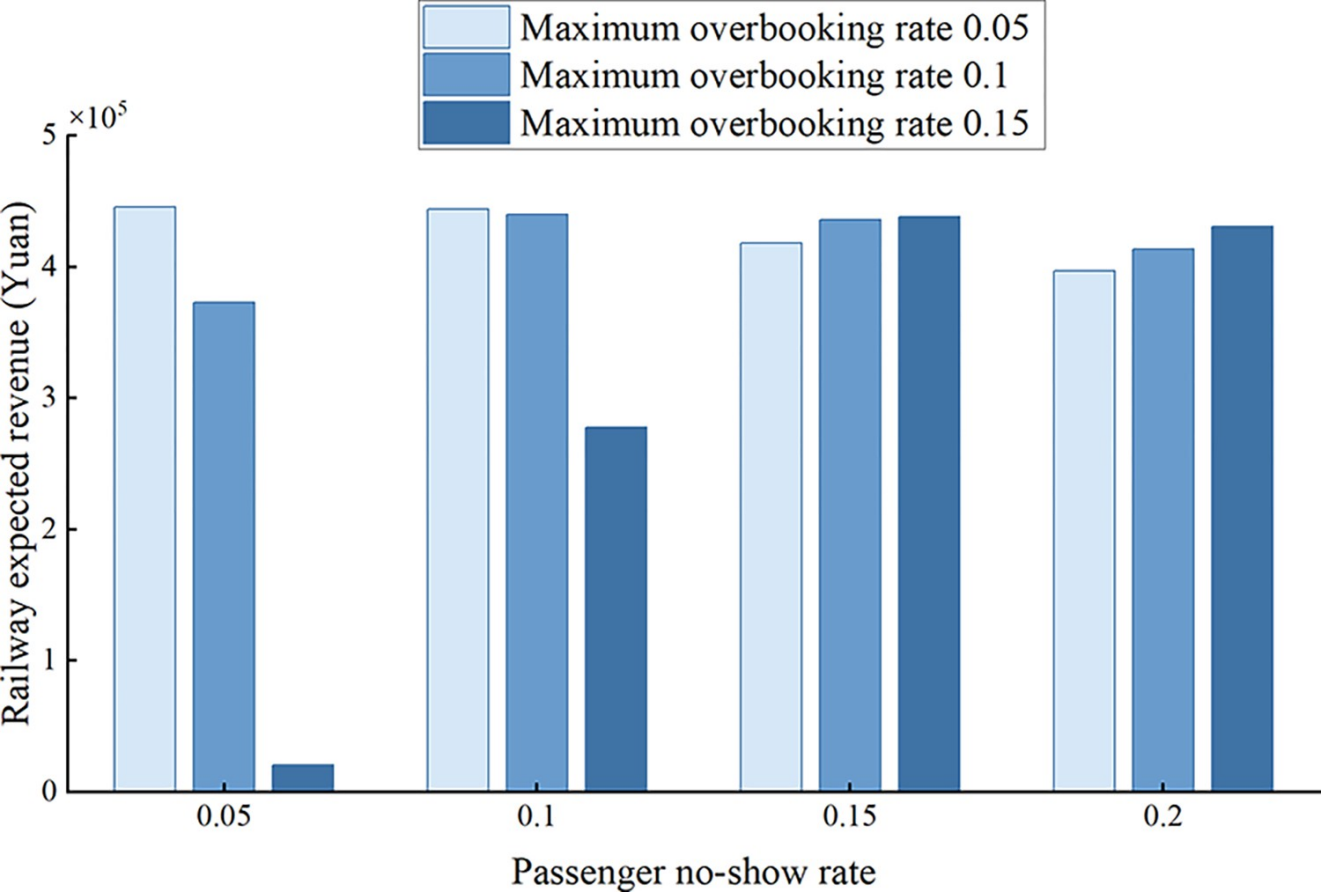
Variation of railway expected revenue with maximum overbooking rate under different passenger no-show rates.

Overbooking is a strategy adopted to deal with passenger no-show behavior and is closely related to passenger no-show rate. When the maximum overbooking rate varies from 0.05 to 0.15 with a step of 0.05, the variation of railway expected revenue with maximum overbooking rate under different passenger no-show rates is shown in Fig 4. It can be seen that the maximum railway expected revenue is generated when the passenger no-show rate and the maximum overbooking rate take similar values. While the maximum overbooking rate is greater than the passenger no-show rate, the railway expected revenue decreases with the maximum overbooking rate. This is because the maximum overbooking rate is too large to produce a large DB cost, which has a negative impact on the railway expected revenue. When the maximum overbooking rate is smaller than the passenger no-show rate, the railway expected revenue rises with the maximum overbooking rate because an increase in the maximum overbooking rate helps to make up for the empty seat loss caused by passenger no-show behavior.

(3) Maximum DB rate

**Fig 5:**
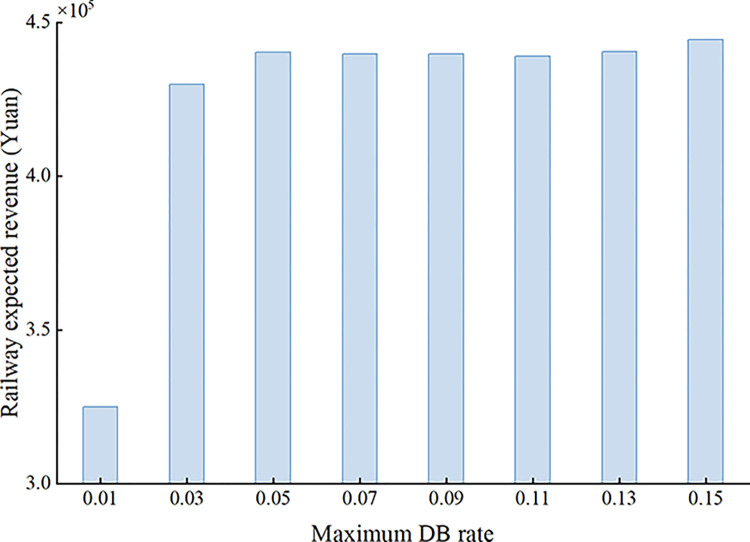
Variation of railway expected revenue with maximum DB rate.

When the maximum DB rate varies from 0.01 to 0.15 with a step of 0.02, the variation of the railway expected revenue with the maximum DB rate is shown in Fig 5. An increase in the maximum DB rate means that more DB is allowed and therefore the railway expected revenue shows an upward trend. An increase in the number of DB passengers also brings about an increase in the DB cost, so the railway expected revenue is relatively stable when the maximum DB rate exceeds 0.05.

(4) DB compensation multiplier

**Fig 6:**
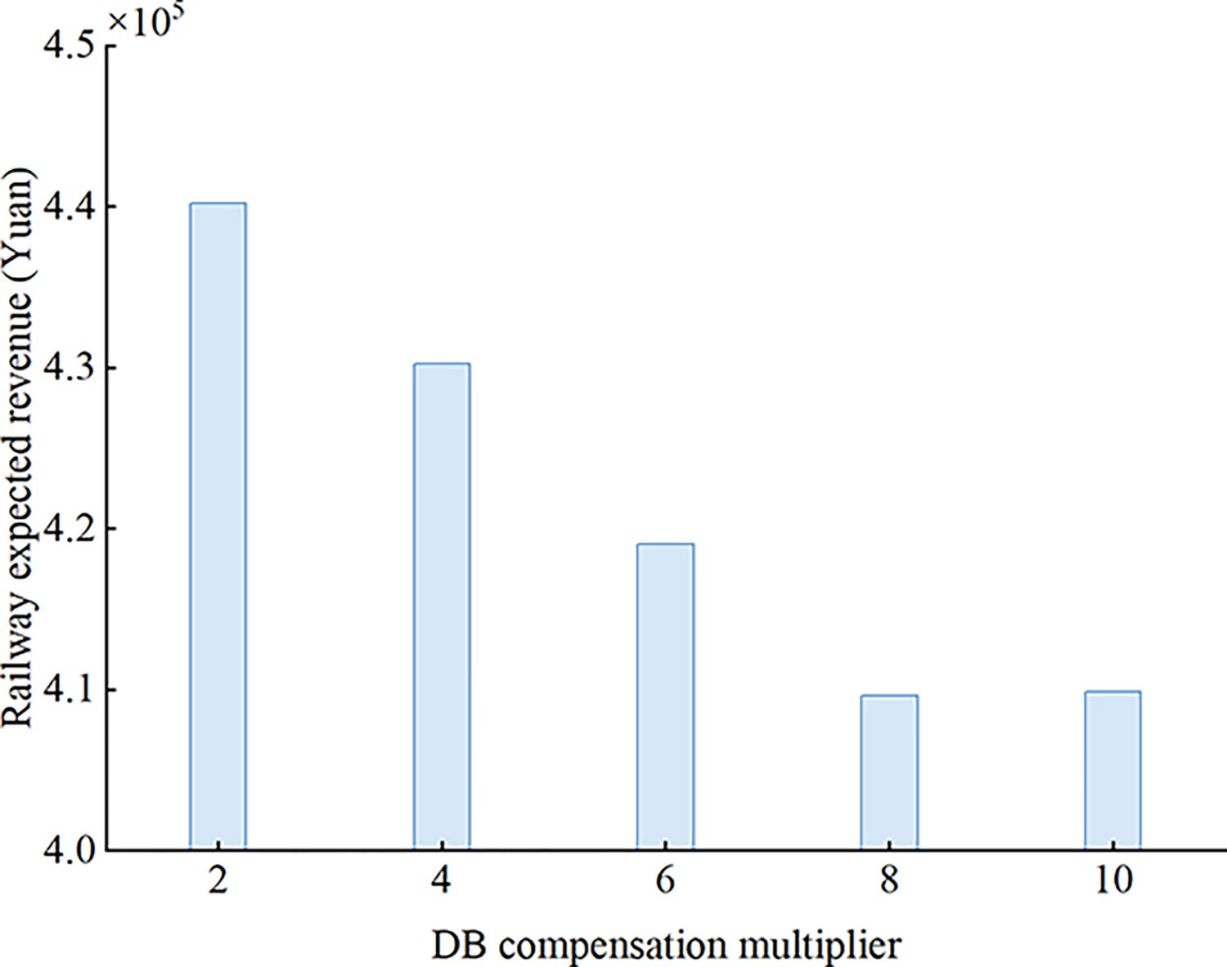
Variation of railway expected revenue with DB compensation multiplier.

When the DB compensation multiplier varies from 2 to 10 with a step of 2, the variation of the railway expected revenue with the DB compensation multiplier is shown in Fig 6. The DB compensation multiplier reflects the unit DB cost per passenger. As the DB compensation multiplier increases, the railway expected revenue gradually decreases.

## 5 Conclusion

To address the influence caused by the uncertainty of passenger demand and passenger no-show behavior on railway revenue and seat allocation, this study investigates the joint optimization problem of overbooking and seat allocation for HSR. Considering train capacity constraints, demand constraints, DB rate constraints and other constraints, a nonlinear stochastic programming model is established with the aim of maximizing the railway expected revenue, and a multi-level optimization method is designed to solve the optimization model. Based on the sampling average approximation method, demand scenarios and passenger no-show scenarios are generated, and the optimization problem is decomposed into the joint optimization problem of overbooking and seat allocation under a single demand scenario and the ticket adjustment problem under other demand scenarios. For the former, it is further decomposed into two sub-problems to be iteratively solved according to the randomness of passenger no-shows. The effectiveness of the proposed model and algorithm is verified by a numerical instance. The results show that the joint optimization method proposed in this paper can effectively deal with the stochasticity of passenger demand and passenger np-show behavior, improving railway revenue and making up for the empty seat loss caused by passenger no-show behavior. In addition, the influences of some important parameters such as passenger no-show rate, maximum overbooking rate, maximum DB rate and DB compensation multiplier on railway revenue are also analyzed. The following conclusions are finally obtained: (1) When the number of demand scenarios and passenger no-show scenarios is 10, a better solution can be obtained within an acceptable calculation time, and after optimization, the railway expected revenue can be increased by about 8.41%; (2) The railway expected revenue generated when the passenger no-show rate and the maximum overbooking rate take similar values is the largest. When the passenger no-show rate is smaller than the maximum overbooking rate, increasing the passenger no-show rate increases the railway expected revenue. When the passenger no-show rate is greater than the maximum overbooking rate, the railway expected revenue decreases with the passenger no-show rate. (3) The railway expected revenue increases with the maximum DB rate and then stabilizes; (4) The railway expected revenue decreases with the DB compensation multiplier.

In addition, passenger demand is elastic affected by factors such as price and travel time in actual operation. For ease of solution, passenger demand is assumed to be stochastic and inelastic in this paper. In future research, passenger demand can be extended to stochastic elastic demand to further study the integrated optimization problem of price, seat allocation and overbooking considering stochastic demand.

## Supporting information

S1 File (ZIP)

## References

[1] Pan L. Operating mileage of China’s railway network hits 159,000 km.; 2024.

[2] Xinhua. China’s railway sector handles record number of passenger trips in 2023.; 2024.

[3] Zhu F, Liu S, Wang R, Wang Z. Assign-to-Seat: Dynamic Capacity Control for Selling High-Speed Train Tickets. Manufacturing & Service Operations Management. 2023; 25(3):921–938. 10.1287/msom.2023.1188.

[4] Yuan W, Nie L. Optimization of seat allocation with fixed prices: An application of railway revenue management in China. PLoS One. 2020; 15(4). doi: 10.1371/journal.pone.0231706 32315337 PMC7173790

[5] Yan Z, Li X, Zhang Q, Han B. Seat allocation model for high-speed railway passenger transportation based on flexible train composition. Computers & Industrial Engineering. 2020; 142:106383. 10.1016/j.cie.2020.106383.

[6] Yan Z, Li X, Han B. Collaborative optimisation of resource capacity allocation and fare rate for high-speed railway passenger transport. Journal of Rail Transport Planning & Management. 2019; 10:23–33. http://doi.org/10.1016/j.jrtpm.2019.05.001.

[7] Xu J, Deng L, Hu X, Wang Q, Zhang Y. Joint Optimization of Multistage Pricing and Seat Allocation for High-Speed Railways Integrating Pre-Sale Period Division. Ieee Transactions On Intelligent Transportation Systems. 2023:1–15. 10.1109/TITS.2023.3324814.

[8] Jiang X, Chen X, Zhang L, Zhang R. Dynamic demand forecasting and ticket assignment for high-speed rail revenue management in China. Transportation Research Record. 2015; 2475(1):37–45. http://doi.org/10.3141/2475-05.

[9] Deng L, Xu J, Zeng N, Hu X. Optimization problem of pricing and seat allocation based on bilevel multifollower programming in high-speed railway. Journal of Advanced Transportation. 2021; 2021:1–15. http://doi.org/10.1155/2021/5316574.

[10] Zhao X, Zhao P. A seat assignment model for high-speed railway ticket booking system with customer preference consideration. Transportmetrica a: Transport Science. 2019; 15(2):776–806. 10.1080/23249935.2018.1532467.

[11] Zhai Q, Tian Y, Luo J, Zhou J. Hotel overbooking based on no-show probability forecasts. Computers & Industrial Engineering. 2023; 180:109226. 10.1016/j.cie.2023.109226.

[12] Xie X, Fan Z, Zhong X. Appointment Capacity Planning With Overbooking for Outpatient Clinics With Patient No-Shows. IEEE Transactions On Automation Science and Engineering. 2022; 19(2):864–883. 10.1109/TASE.2021.3060567.

[13] Wang T, Xing Z, Hu H, Qu X. Overbooking and delivery-delay-allowed strategies for container slot allocation. Transportation Research Part E: Logistics and Transportation Review. 2019; 122:433–447. 10.1016/j.tre.2018.12.019.

[14] Sun H, Lam JSL, Zeng Q. The dual-channel sales strategy of liner slots considering shipping e-commerce platforms. Computers & Industrial Engineering. 2021; 159:107516. 10.1016/j.cie.2021.107516.

[15] Qasim SM, Farooquie JA. Economics of secondary queue of Indian railways passenger reservation system: a queueing science approach. Opsearch. 2024. 10.1007/s12597-024-00755-3.

[16] Fard FA, Sy M, Ivanov D. Optimal overbooking strategies in the airlines using dynamic programming approach in continuous time. Transportation Research Part E: Logistics and Transportation Review. 2019; 128:384–399. 10.1016/j.tre.2019.07.001.

[17] Leff G. New DOT Rule Raises Involuntary Denied Boarding And Mishandled Bag Compensation.; 2021.

[18] Chatwin RE. Continuous-time airline overbooking with time-dependent fares and refunds. Transportation Science. 1999; 33(2):182–191. http://doi.org/10.1287/trsc.33.2.182.

[19] Shlifer E, Vardi YJTS. An airline overbooking policy. Transportation Science. 1975; 9(2):101–114. http://doi.org/10.1287/trsc.9.2.101.

[20] Huang Y, Ge Y, Zhang X, Xu Y. Overbooking for parallel flights with transference. International Journal of Production Economics. 2013; 144(2):582–589. http://doi.org/10.1016/j.ijpe.2013.04.021.

[21] Dalalah D, Ojiako U, Chipulu M. Voluntary overbooking in commercial airline reservations. Journal of Air Transport Management. 2020; 86:101835. 10.1016/j.jairtraman.2020.101835.

[22] Lan Y, Ball MO, Karaesmen IZ. Regret in Overbooking and Fare-Class Allocation for Single Leg. Manufacturing & Service Operations Management. 2011; 13(2):194–208. 10.1287/msom.1100.0316.

[23] Lan Y, Ball MO, Karaesmen IZ, Zhang JX, Liu GX. Analysis of seat allocation and overbooking decisions with hybrid information. European Journal of Operational Research. 2014. 10.1016/j.ejor.2014.07.021.

[24] Guo S. Discuss of China’s high speed railway overbooking. Railway Transport and Economy. 2008; 30(8):87–89. 10.3969/j.issn.1003-1421.2008.08.030.

[25] Yan R, Feng T, Tian Z. Research on the overbooking problem of passenger dedicated line in China. In: Editor, editor In ICLEM 2010: Logistics for Sustained Economic Development: Infrastructure, Information, Integration; 2010. Pub Place; 2010.

[26] Sato K, Sawaki K. Dynamic pricing of high-speed rail with transport competition. Journal of Revenue and Pricing Management. 2012; 11:548–559. http://doi.org/10.1057/rpm.2011.29.

[27] Deng L, Xu J, Peng Q, Hu X. Overbooking strategy analysis of high-speed train based on stochastic programming. Journal of Railway Science and Engineering. 2022; 19(10):2813–2819. 10.19713/j.cnki.43-1423/u.t20211294.

[28] Wang X, Wang H, Zhang X. Stochastic seat allocation models for passenger rail transportation under customer choice. Transportation Research Part E: Logistics and Transportation Review. 2016; 96:95–112. 10.1016/j.tre.2016.10.003.

